# Treatment-related changes in neuroendocrine tumors as assessed by textural features derived from ^68^Ga-DOTATOC PET/MRI with simultaneous acquisition of apparent diffusion coefficient

**DOI:** 10.1186/s12885-020-06836-y

**Published:** 2020-04-16

**Authors:** Manuel Weber, Lukas Kessler, Benedikt Schaarschmidt, Wolfgang Peter Fendler, Harald Lahner, Gerald Antoch, Lale Umutlu, Ken Herrmann, Christoph Rischpler

**Affiliations:** 1 Department of Nuclear Medicine, University Hospital Essen, University of Duisburg-Essen, Essen, Germany; 2 Institute of Diagnostic and Interventional Radiology and Neuroradiology, University Hospital Essen, University Duisburg-Essen, Essen, Germany; 3 Department of Endocrinology and Metabolism, Division of Laboratory Research, University Hospital Essen, University Duisburg-Essen, Essen, Germany; 4 Department of Diagnostic and Interventional Radiology, Medical Faculty, Heinrich Heine University Düsseldorf, Düsseldorf, Germany

**Keywords:** DOTATOC, PET/MRI, Textural features, Radiomics, NET, Radiopeptide therapy

## Abstract

**Background:**

Neuroendocrine tumors (NETs) frequently overexpress somatostatin receptors (SSTRs), which is the molecular basis for ^68^Ga-DOTATOC positron-emission tomography (PET) and radiopeptide therapy (PRRT). However, SSTR expression fluctuates and can be subject to treatment-related changes. The aim of this retrospective study was to assess, which changes in PET and apparent diffusion coefficient (ADC) occur for different treatments and if pre-therapeutic ^68^Ga-DOTATOC-PET/MRI was able to predict treatment response to PRRT.

**Methods:**

Patients with histopathologically confirmed NET, at least one liver metastasis > 1 cm and at least two ^68^Ga-DOTATOC-PET/MRI including ADC maps were eligible. ^68^Ga-DOTATOC-PET/MRI of up to 5 liver lesions per patients was subsequently analyzed. Extracted features comprise conventional PET parameters, such as maximum and mean standardized uptake value (SUVmax and SUVmean) and ADC values. Furthermore, textural features (TFs) from both modalities were extracted. In patients with multiple ^68^Ga-DOTATOC-PET/MRI a pair of 2 scans each was analyzed separately and the parameter changes between both scans calculated. The same image analysis was performed in patients with ^68^Ga-DOTATOC-PET/MRI before PRRT. Differences in PET and ADC maps parameters between PRRT-responders and non-responders were compared using Mann-Whitney test to test differences among groups for statistical significance.

**Results:**

29 pairs of ^68^Ga-DOTATOC-PET/MRI scans of 18 patients were eligible for the assessment of treatment-related changes. In 12 cases patients were treated with somatostatin analogues between scans, in 9 cases with PRRT and in 2 cases each patients received local treatment, chemotherapy and sunitinib. Treatment responders showed a statistically significant decrease in lesion volume and a borderline significant decrease in entropy on ADC maps when compared to non-responders. Patients treated with standalone SSA showed a borderline significant decrease in mean and maximum ADC, compared to patients treated with PRRT. No parameters were able to predict treatment response to PRRT on pre-therapeutic ^68^Ga-DOTATOC-PET/MRI.

**Conclusions:**

Patients responding to current treatment showed a statistically significant decrease in lesion volume on ADC maps and a borderline significant decrease in entropy. No statistically significant changes in PET parameters were observed. No PET or ADC maps parameters predicted treatment response to PRRT. However, the sample size of this preliminary study is small and further research needed.

## Background

Even though neuroendocrine neoplasias (NENs) fall among the less frequent neoplasms in humans a fivefold increase in incidence over the past decades has been observed (1973: 1.09/100,000: 2004, 5.25/100,000) making advances both in treatment and diagnostics much needed [1]. They derive from the diffuse endocrine system of a variety of organs, with the most frequent primary sites being pancreas, gastrointestinal tract, lungs and the thymus [2]; in approximately 13% the primary remains unknown [3]. The heterogeneity of NENs also extends to their level of differentiation: it can vary from well-differentiated tumors with a low proliferative activity to undifferentiated neuroendocrine carcinomas (NECs) with a very high proliferative activity and grim prognosis [2]. Well differentiated NENs are known to overexpress somatostatin receptors (SSTRs), which can be used diagnostically by virtue of ^68^Ga-DOTATOC positron-emission tomography (^68^Ga-DOTATOC-PET) [1]. Hybrid imaging with ^68^Ga-DOTATOC-PET and computed tomography (CT) or magnetic resonance imaging (MRI) has shown superior diagnostic performance when compared to standalone CT or MRI [4, 5]. PET/MRI allows for the simultaneous acquisition of diffusion-weighted MRI (DW-MRI) and has additional advantages over PET/CT with regards to the detection of liver metastases [6–8].

DW-MRI visualizes the motion of water molecules in the intracellular, intravascular and extracellular space; changes in water motion are observed particularly in hypercellular tumors due to diffusion restriction. This process can be quantified by apparent diffusion coefficient (ADC) maps [9]. It was shown to be a valid modality to assess treatment response in different tumor entities, such as prostate cancer, glioma, malignant liver tumors and breast cancer [10–12].

Accurate diagnostic workup in patients with NET is of particular interest, especially as up to 85% of patients with NET present with distant metastases at initial diagnosis [13–15]; in this setting oftentimes resection with curative intent is not feasible and systemic treatment is performed instead:

In the context of well-differentiated NETs long-acting somatostatin analogs (SSA) have been shown to achieve symptom control and prolongation of progression-free survival (PFS) and are chosen as first line treatment, especially in hormonally active NENs [3, 16–18]. After disease progression on treatment with SSA occurs, a variety of second-line treatments can be initiated:

In pancreatic NET (pNET) Everolimus and different chemotherapy regiments [19, 20] have shown convincing results [21–28]. Furthermore, the NETTER-1 trial has shown increased PFS and OS in patients with midgut NET treated with 4 cycles of Lu-177-DOTATATE as second line treatment [29]. It has already been shown that early changes in SSTR expression, as assessed by ^68^Ga-DOTATATE-PET/CT correlate with time to progression and clinical symptoms in patients receiving radiopeptide therapy (PRRT), making molecular imaging via PET a suitable modality to assess treatment response in NET patients [30]. Furthermore, there are studies indicating that ^68^Ga-DOTATOC-PET/CT might be able to predict treatment response to PRRT [31]. In this context large scale feature extraction, also referred to as radiomics may be able to identify underlying patterns that remain unnoticed by the human eye and provide additional information about the examined pathology: Prior studies in the field of radiomics in PET imaging have shown that, for instance, tumor heterogeneity is a negative prognostic marker that decreases in patients responding to their current treatment [32–34].

As the therapeutic effect of all of the aforementioned systemic treatments is mitigated via different biological pathways, changes in SSTR expression might deviate from one another. The aim of this study was to examine if different therapeutic agents induce different changes in SSTR expression as assessed by a subset of textural features (TFs) derived from ^68^Ga-DOTATOC-PET/MRI and ADC maps. Additionally, we evaluated the predictive value of these TFs in patients undergoing ^68^Ga-DOTATOC-PET/MRI before PRRT with regards to therapy response.

## Methods

### Eligibility criteria

315 DOTATOC-PET/MRI scans were screened for the following criteria:

- Histopathologically confirmed NET
- At least one liver lesion with > 1 cm in size in both scans
- ^68^Ga-DOTATOC-PET/MRI scans were performed with simultaneous acquisition of ADC maps
- *Either:*^68^Ga-DOTATOC-PET/MRI was performed before PRRT and follow-up imaging was available. This cohort was used to evaluate the predictive value of pre-PRRT ^68^Ga-DOTATOC-PET/MRI for treatment response.
- *Or:* A follow-up ^68^Ga-DOTATOC-PET/MRI was available and the treatment between the scans documented. This cohort was used to compare treatment-related changes among patients undergoing different types of treatment and between responders and non-responders. Therapy response was defined as complete response, partial response and stable disease, while no therapy response was defined as progressive disease, both according to RECIST 1.1 criteria [35]. In patients with multiple examinations that met the aforementioned criteria the follow-up scan was also used as baseline scan for the subsequent examination. Hence, in patients with 3 ^68^Ga-DOTATOC-PET/MRI scans, changes between the first and second, and between the second and third scan were analyzed separately. In patients with 4 ^68^Ga-DOTATOC-PET/MRI scans changes between the first and second, second and third, third and fourth scan were evaluated individually, etc. While PRRT was strictly performed after the baseline scan, in patients undergoing SSA, chemotherapy and other systemic treatments the respective therapy had already been initiated at the time of the baseline scan.

### Image acquisition

Whole-body (i.e. skull base to mid-thigh) ^68^Ga-DOTATOC-PET/MRI was performed on an integrated 3.0 T Biograph mMR scanner (Siemens Healthcare GmbH) in accordance with published guidelines [36, 37]. Patients were not required to fast; discontinuing therapy with long-acting somatostatin analogs was encouraged but not mandatory as there is no effect on tracer uptake in neuroendocrine tumors [38]. In the study cohort with ^68^Ga-DOTATOC-PET/MRI before PRRT on average 65 (range: 44–78) Megabecquerel were administered intravenously. This relatively low activity can be explained by the higher sensitivity of the used PET/MRI compared to PET/CT [39].

After a mean interval of 102 min (range: 30–120) the image acquisition was started. In the study cohort with follow-up by virtue of ^68^Ga-DOTATOC-PET/MRI on average 71 (range: 40–121) Megabecquerel ^68^Ga-DOTATOC were administered and image acquisition was started after an interval of 85 (range: 30–180) minutes.

All PET/MRIs were performed with a Siemens mMR Biograph, as published elsewhere [8].

### Image analysis

**Fig. 1:**
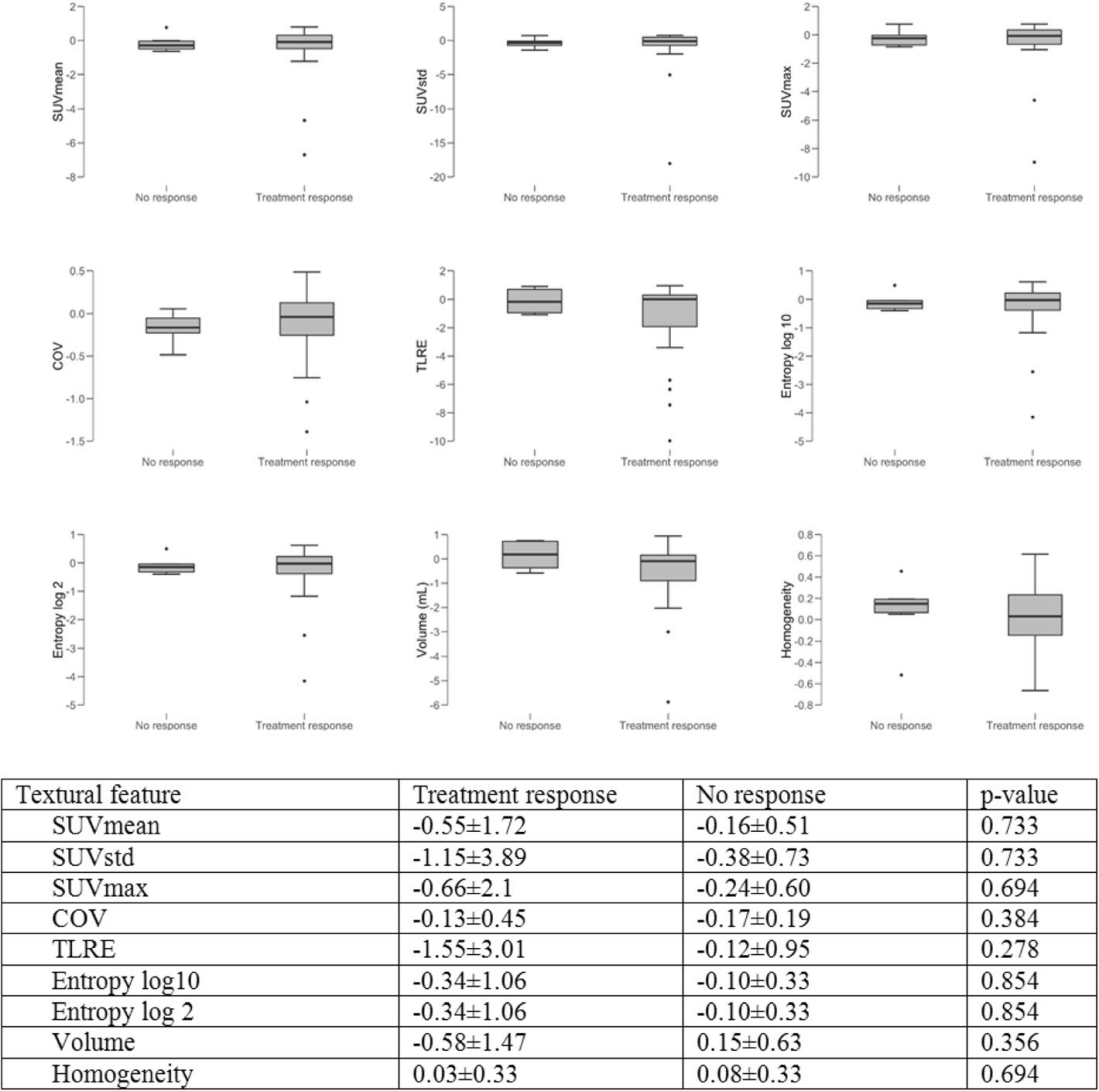
Boxplots and charts displaying **c**hanges in DOTATOC-PET parameters, when comparing patients showing treatment response to their current treatment with patients with disease progression

**Fig. 2:**
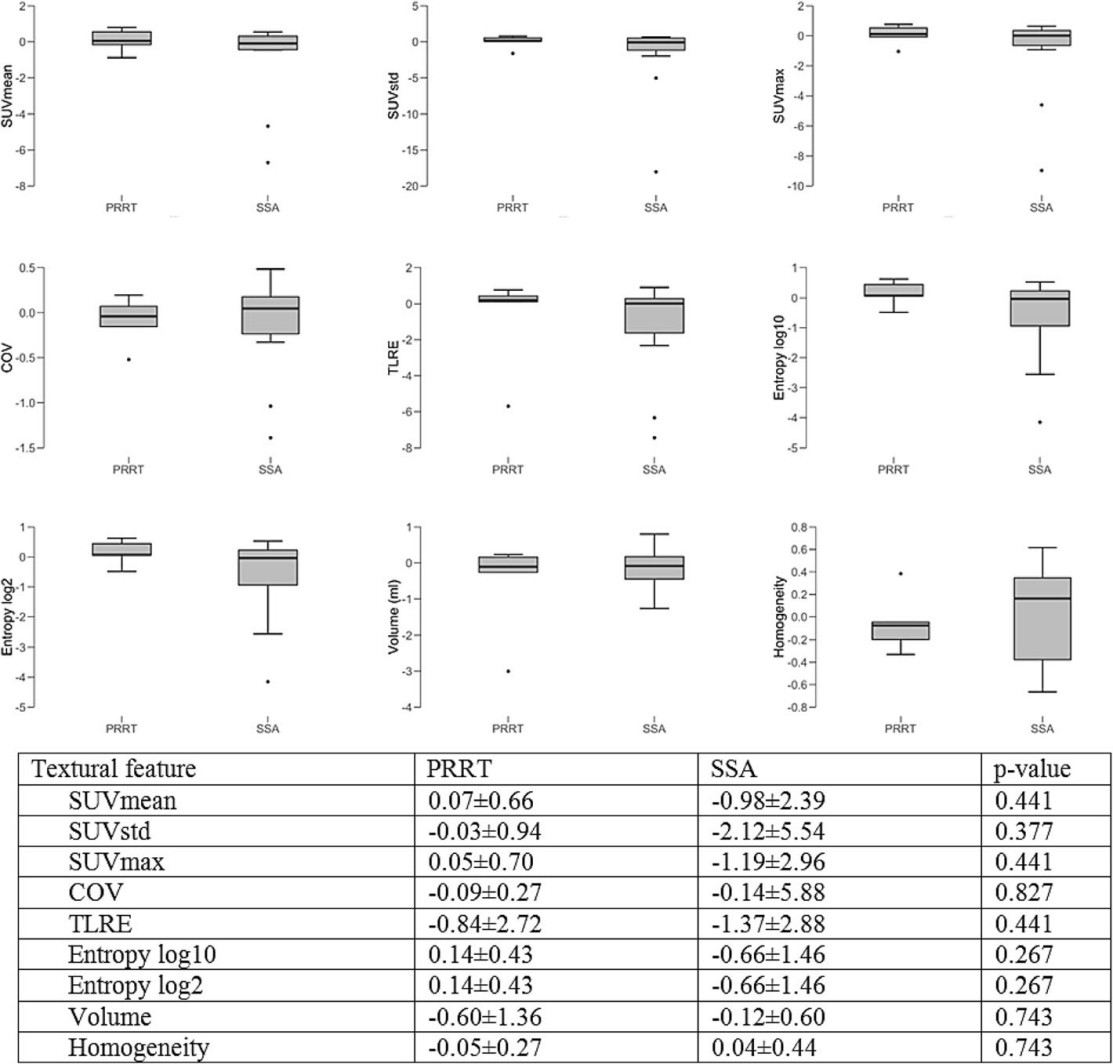
Boxplots and charts displaying changes DOTATOC-PET parameters, when comparing patients undergoing SSA vs. patients undergoing PRRT

**Fig. 3:**
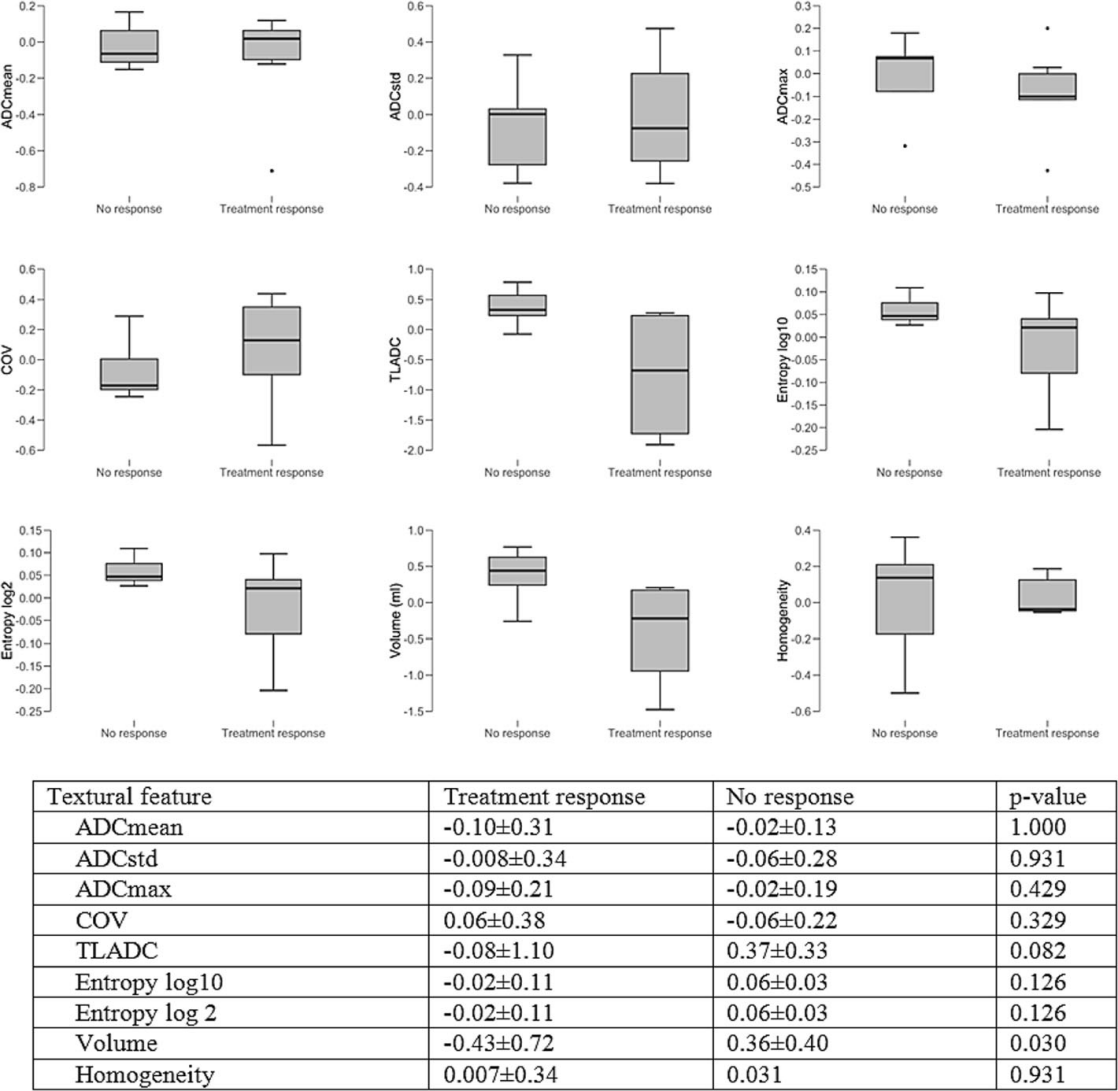
Boxplots and charts showing changes in ADC map, when comparing patients showing treatment response to their current treatment with patients with disease progression

**Fig. 4:**
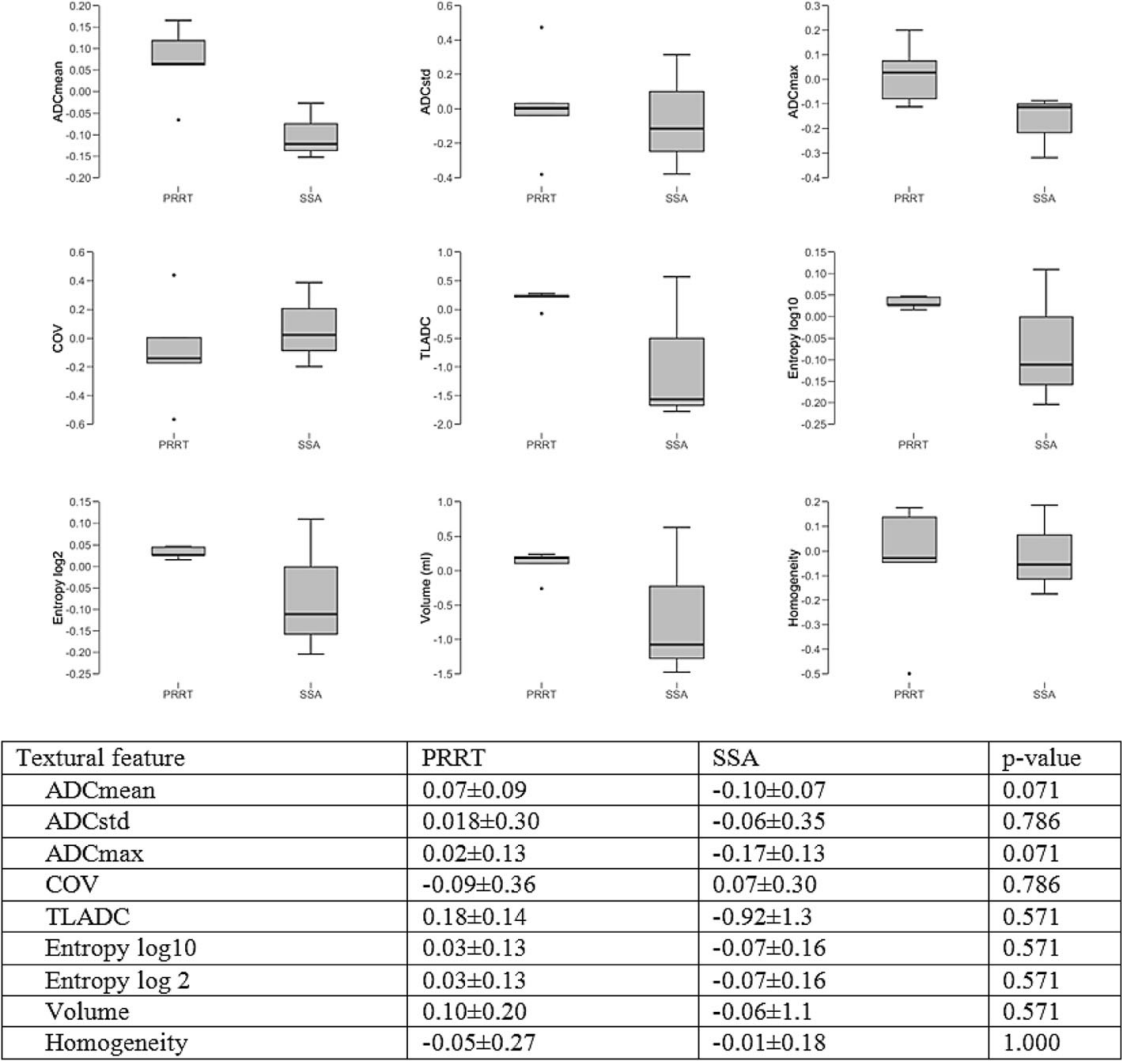
Boxplots and charts displaying changes in ADC map, when comparing patients undergoing SSA vs. patients undergoing PRRT

**Fig. 5:**
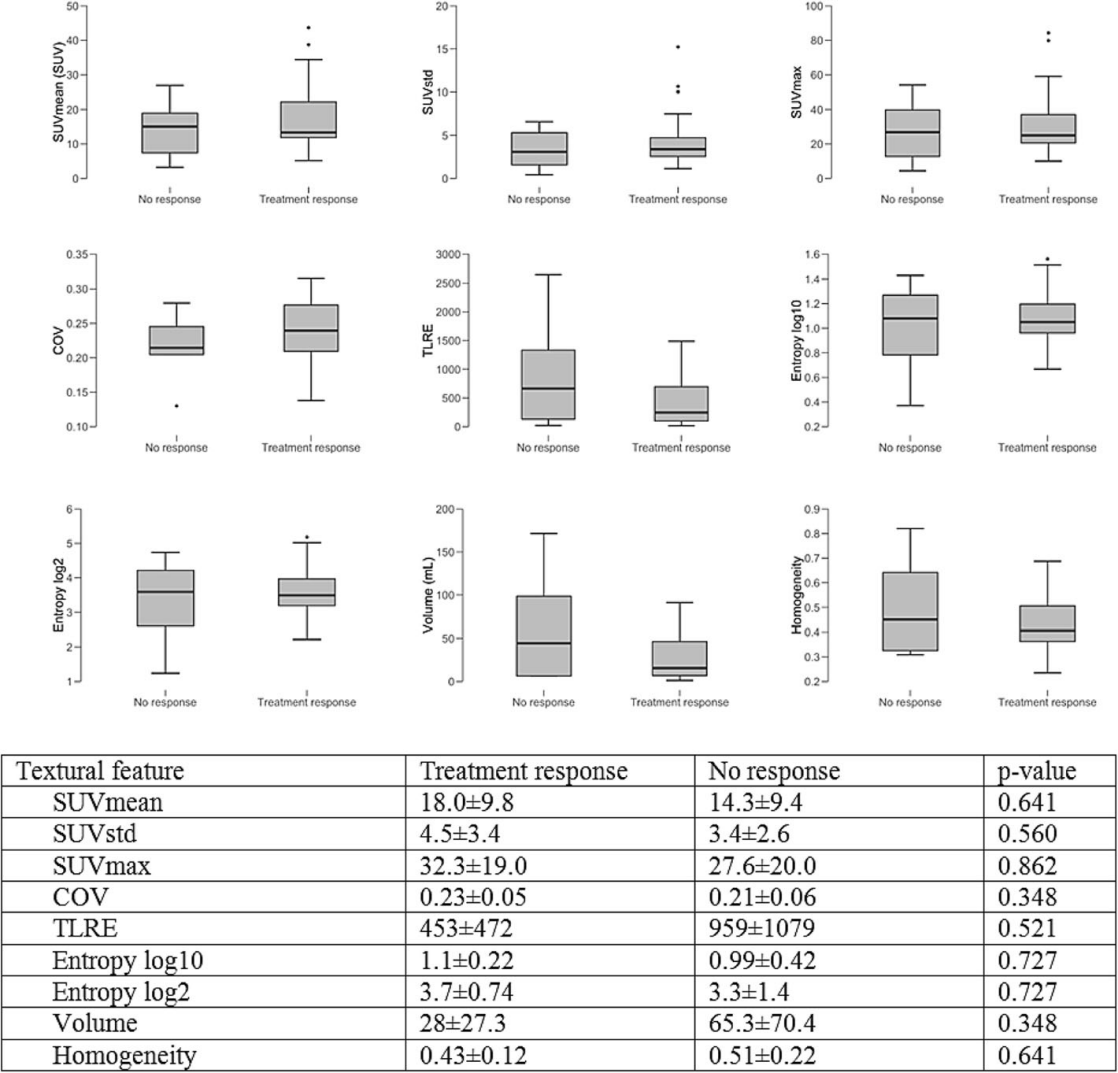
Boxplots and charts showing the differences in pre-PRRT SSTR expression as assessed by 68Ga-DOTATOC-PET/MRI between patients who would later respond to treatment and patients who would not. SUVmean, mean standardized uptake value; SUVstd, standard deviation of standardized uptake value; SUV max, maximum standardized uptake value; cov, coefficient of variance; TLRE, total lesion receptor expression\

**Fig. 6:**
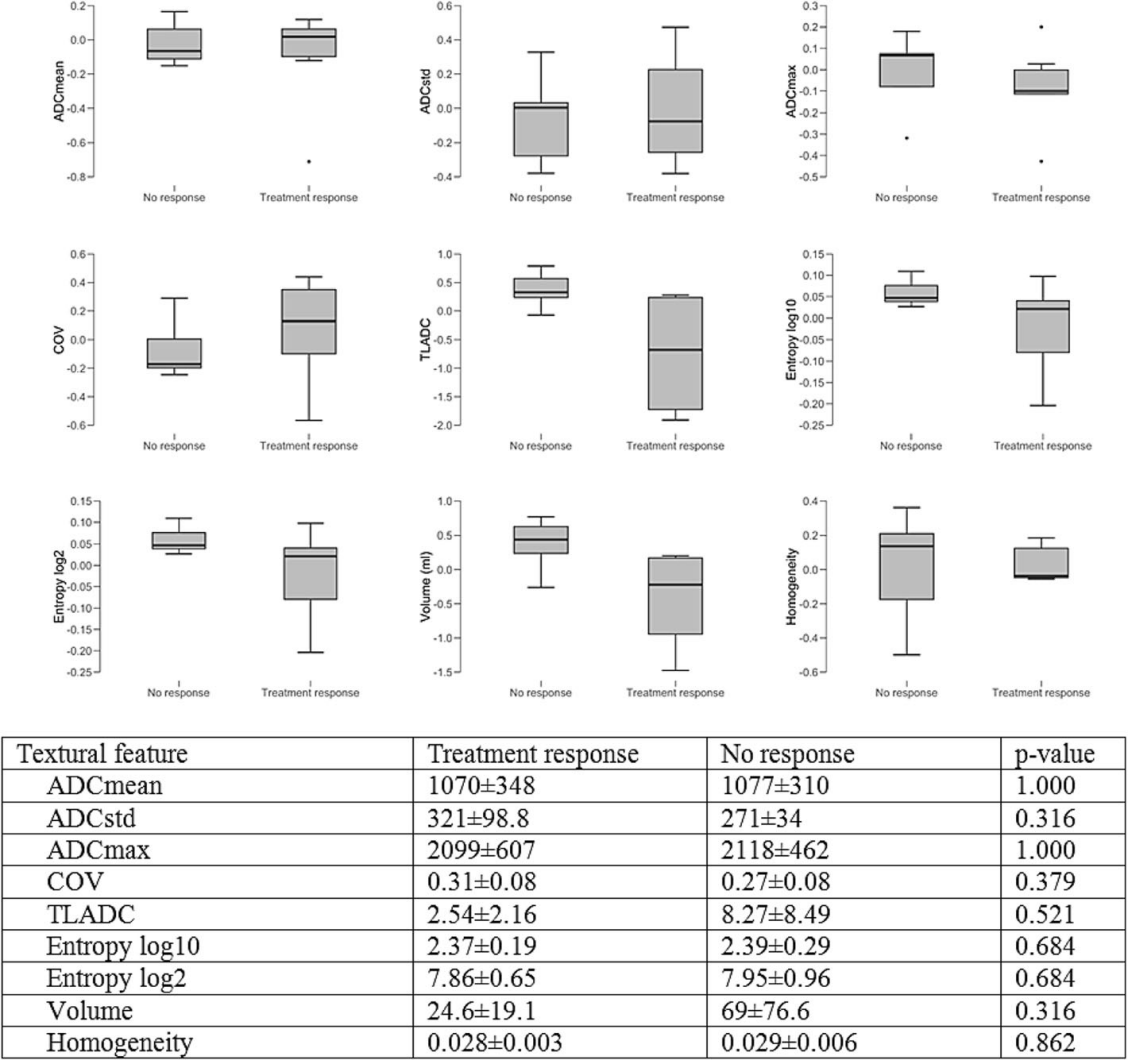
Boxplots and charts showing the differences in pre-PRRT ADC maps between patients who would later respond to treatment and patients who would not. ADCmean, mean apparent diffusion coefficient; ADCstd, standard deviation of apparent diffusion coefficient; ADCmax, maximum apparent diffusion coefficient; COV, coefficient of variance; TLADC, total lesion apparent diffusion coefficient

Image analysis was performed using LIFEx, as follows [40]:

For PET the tumor margins of liver metastases were delineated in every slice using the region of interest (ROI) tool, encompassing photopenic, cystic and necrotic regions within the tumor. This procedure was performed for up to 5 liver lesions per patient; in patients with more than five lesions the 5 largest lesions were selected. If possible, tumor delineation was performed semi-automatically by choosing a 40% threshold. However, this was not possible in some lesions with very faint / missing SSTR-expression and manual segmentation based on the coregistered MRI was performed instead. SUV values between 0 and 150, which entails the maximum SUV value in the study cohort, where rescaled with a fixed bin width of 1 and feature extraction was executed. The same procedure was then performed for the same lesions on the follow-up scan. For texture analysis of ADC maps, ROI delineation was performed visually, again encompassing cystic and necrotic regions. In this case relative intensity rescaling with mean + − 3 standard deviations and a fixed number of grey levels (1000) were used. An overview of the assessed TFs is given in Figs. 1, 2, 3, 4, 5 and 6. Supplemental Figs. 1 and 2 show ADC maps and DOTATOC-PET images of baseline and follow-up scans of two exemplary patients.

### Statistical analysis

Statistical analysis was performed using JASP (version 0.9.2.0). For each patient the mean of the assessed TFs across all measured lesions was calculated to avoid overrepresentation of data gathered from patients with multiple lesions compared to patients with only one or few assessed hepatic lesion.

In patients, where follow-up ^68^Ga-DOTATOC-PET/MRI was available, the changes in TFs from ^68^Ga-DOTATOC-PET and ADC maps were calculated and compared between patients undergoing SSA therapy vs. PRRT and between patients showing therapy response vs. no therapy response.

Finally, Mann-Whitney test was employed to test differences in TFs among groups for statistical significance, with a *p*-value < 0.05 being considered statistically significant.

In patients, in whom ^68^Ga-DOTATOC-PET/MRI was performed before PRRT the mean of all TFs derived from ^68^Ga-DOTATOC-PET and ADC maps was calculated and compared between patients with therapy response vs. no therapy response. Statistical significance in this cohort was assessed as outlined above.

## Results

### Treatment-related changes

#### Patients’ characteristics

A total of 18 different patients met the inclusion criteria and were included into this study. Of these, in 4 patients with pancreatic NET and 2 patients with small intestinal NET 3 ^68^Ga-DOTATOC-PET/MRI scans each were available. In 1 patient with pancreatic NET 4 ^68^Ga-DOTATOC-PET/MRI scans were available and in 1 patient with small intestinal NET 5 ^68^Ga-DOTATOC-PET/MRI scans were available, amounting to a total of 29 baseline/follow-up pairs.

Mean patient age for each patient (baseline/follow-up couple) at the time of baseline scan was 58.6 (58.0) years. 8 (6) patients were female, 21 (12) male.

Of the 18 assessed patients (29 baseline/follow-up couples) 11 (17) had NET of the pancreas, 5 (10) had NET of the small intestine and 1 (1) patient each had NET of the lung and kidney. 5 (9) of the patients had low-grade NET, 10 (14) had intermediate-grade and 3 (6) had high-grade NET. Average time between baseline and follow-up ^68^Ga-DOTATOC-PET/MRI was 371 (interquartile range: 418) days.

A detailed overview over the patients is provided in Table 1.

**Table 1:** Characteristics of baseline/follow-up pairs and the respective patients, who underwent ^68^Ga-DOTATOC-PET/MRI at baseline and follow-up and who were analyzed to assess treatment-related changes

	Baseline/ follow-up pairs (***N*** = 29)	Patients (***N*** = 18)
**Age (years)**		
Median (range)	58.6 (18–82)	58.0 (18–82)
**Sex**	N	N
Male	21	12
Female	8	6
**Primary**	N	
Pancreas	17	11
Small intestine	10	5
Lungs	1	1
Kidney	1	1
**Grading**	N	
1	9	5
2	14	10
3	6	3
**Treatment response**		
Treatment response	23	
No response	6	
**Systemic treatment**		
SSA	13	
PRRT	10	
Chemotherapy	2	
Sunitinib	2	
None	2	

#### Treatment-related changes in PET parameters

80 lesions were segmented and the following parameters assessed: SUVmean, SUVstd, SUVmax, COV, TLRE, Entropy log10, Entropy log 2, Volume, Homogeneity. These features were selected based on prior studies showing their predictive value with regards to treatment response and histopathology [41–45].

23/29 (79%) patients responding to their current treatment protocol showed a tendency towards a larger decrease (− 1.55 ± 3.01 vs. -0.12 ± 0.95 in total lesion receptor expression. However, the difference between responders and non-responders was not statistically significant (*p* = 0.278). Patients undergoing SSA therapy alone tended to show a decrease in entropy (− 0.07 ± 0.16) when compared to patients undergoing PRRT (0.14 ± 0.43). In the latter, PRRT and the last administration of long-acting somatostatin analogues before PRRT were scheduled 4 weeks apart similar to the NETTER-1 protocol [29]. However, differences among groups did not reach statistical significance, either (*p* = 0.267). A detailed overview over the assessed treatment-related changes in SSTR-expression is provided in Figs. 1 and 2.

#### Treatment-related changes in ADC values

As not all PET-positive lesions showed a sharp delineation, and the segmentation of those lesions would have encompassed physiological liver tissue, 26 well-defined lesions were chosen for segmentation and the following parameters extracted: ADCmean, ADCstd, ADCmax, COV, TLADC, Entropy log10, Entropy log 2, Volume, Homogeneity: 7/13 (54%) patients responding to current treatment showed a statistically significant decrease in the lesion volume on ADC maps (− 0.43 ± 0.72 vs. 0.36 ± 0.40; *p* = 0.030). All other changes in diffusion restriction did not reach statistical significance. Patients treated with SSA showed a decrease in ADC mean (− 0.10 ± 0.07) and ADC max (− 0.17 ± 0.13) when compared to patients receiving PRRT (ADC mean: 0.07 ± 0.09; ADC max: 0.02 ± 0.13); differences among groups were of borderline statistical significance (*p* = 0.071). There were no statistically significant differences for the other TFs when comparing patients treated with SSA and patients treated with PRRT. One patient with progressive disease showed new lesions on ADC maps. Vice versa one patient with treatment response showed a disappearance of lesions on ADC maps.

### Predictive value of pre-therapeutic ^68^Ga-DOTATOC-PET/MRI with regards to PRRT response

The predictive value of pre-therapeutic DOTATOC-PET/MRI was assessed for PRRT only, as the patient numbers for the other therapies were too low.

#### Patient characteristics

A total of 28 patients met the inclusion criteria. Mean patient age was 66.1 years (22.4–88.0). Treatment response was observed in 23 cases, in 5 cases disease progression despite PRRT occurred. In 10 cases the primary was located in the pancreas, in 9 in the small intestine and unknown in 4 cases. 9 patients had low-grade, 15 intermediate-grade and 3 high-grade tumors; grading was not assessed in one patient with paraganglioma, as there were no established grading systems at the time of initial diagnosis [46]. 3/28 patients were treated according to the NETTER-1 protocol. 15 patients received ^177^Lu-DOTATOC, 13 ^90^Yttrium-DOTATOC. A detailed overview over the patients’ characteristics is provided in Table 2. 90 PET lesions and 57 ADC lesions were analyzed.

**Table 2:** Characteristics of patients with follow-up, who underwent ^68^Ga-DOTATOC-PET/MRI before PRRT and were used to assess the predictive value of ^68^Ga-DOTATOC-PET/MRI

All patients (***N*** = 28)	
**Age (years)**	
Median (range)	66.1 (22–88)
**Sex**	N
Male	17
Female	11
**Primary**	N
Pancreas	10
Small intestine	9
CUP	4
Lungs	1
Rectum	1
Stomach	1
Kidney	1
Paraganglioma	1
**Grading**	N
1	9
2	14
3	3
Unknown	
**Treatment response**	
Treatment response	23
No response	5
**Nuclide**	
^177^Lutetium	15
^90^Yttrium	13
**NETTER-1 protocol**	
yes	3
no	25
**Cycles before follow-up**	
1	24
2	1
4	3
**Therapeutic Activity**	
Mean (range)	7.92 (4–30.2)

#### Textural features derived from PET in patients with pre-PRRT ^68^Ga-DOTATOC-PET/MRI

Mean SUV mean was 17.95 ± 9.8 in patients, who showed a therapy response to PRRT and 14.3 ± 9.4 in patients with disease progression despite PRRT (*p* = 0.641). Mean SUV max was 32.3 ± 19.0 in patients with therapy response and 27.6 ± 20.0 in patients without therapy response (*p* = 0.862). SUVstd, TLRE, Entropy log10, Entropy log 2, Volume and Homogeneity didn’t show statistically significant differences, either. A detailed overview over the assessed TFs and statistical significance is provided in Fig. 5.

#### Textural features derived from ADC map in patients with pre-PRRT ^68^Ga-DOTATOC-PET/MRI

Mean ADC mean was 1070 ± 348 in patients, who would later show a treatment response and 1077 ± 310 in patients with no treatment response (*p* = 1.000). Mean ADC max was 2099 ± 607 (*p* = 0.316) in patients who subsequently responded to PRRT and 2118 ± 462 in patients who would not. Statistically significant differences were not observed for ADCstd, COV, TLADC, Entropy log10, Entropy log 2, Volume, Homogeneity between groups, either.

## Discussion

In this preliminary retrospective study, we showed that patients showing treatment response experienced statistically significant reduction of tumor volume on ADC maps and borderline significant reduction of total lesion apparent diffusion coefficient when compared to patients with disease progression. Patients responding to treatment showed a decrease in entropy of ADC maps lesions as well, although statistical significance was not reached (*p* = 0.126). Furthermore, patients undergoing treatment with SSA tended to show a decrease in ADCmean and ADC maps, which was borderline significant (*p* = 0.071) when compared to patients treated with PRRT. However, this might be a result of the small sample size (*n* = 11 for treatment response vs. no response; *n* = 8 for SSA vs. PRRT). There were no statistically significant differences in PET parameters.

To our knowledge there are no studies examining treatment-related changes on ^68^Ga-DOTATOC-PET/MRI as assessed by conventional PET and ADC parameters as well as textural features. However, treatment-related changes in FDG-PET are well researched and a reduction in FDG-uptake has been shown to be an early sign of treatment response in different tumor entities, when changes on morphological imaging have not occurred yet [47–49]. Furthermore, prior studies on other tumor entities have shown the potential of PET to predict treatment response: Lue et al. showed that higher tumor heterogeneity as assessed by FDG-PET was negatively correlated with overall survival in the context of Hodgkin lymphoma [50] In another study, Huang et al. showed that total lesion glycolysis at baseline was negatively correlated with treatment response in patients with non-small-cell lung cancer [51].

In our study, however, we were not able to reproduce most of these findings in NETs using hybrid ^68^Ga-DOTATOC-PET/MRI. No statistically significant differences PET- and ADC-wise were observed, when comparing patients who received a ^68^Ga-DOTATOC-PET/MRI before treatment with PRRT. This is in contrast to studies by Öksüz et al. and Werner et al., who showed statistically significant differences between responders and non-responders with regards to conventional SUV parameters and tumor heterogeneity as assessed by ^68^Ga-DOTATOC-PET [31, 52]. In contrast to both studies we assessed conventional PET and ADC maps parameters exclusively from liver lesions, as the presence or absence of liver metastases has been shown to be a major prognostic factor [53, 54]. Additionally, Werner et al., only included G1 and G2 pancreatic NETs, while we included NETs of all gradings and primaries.

The difference in result might also be explained by the low amount of non-responders in our study cohort (*n* = 5 for PET analysis; *n* = 4 for ADC analysis). A further possible confounder is that the patients in our study cohort were not treated following the same protocol, with some of them being treated with ^177^Lutetium-DOTATOC, others with ^90^Yttrium-DOTATOC; some underwent multiple cycles between baseline DOTATOC-PET/MRI and assessment of treatment response, others underwent one cycle. Time interval between baseline PET/MRI and assessment of treatment response was heterogeneous. However, none of these studies were performed by virtue of ^68^Ga-DOTATOC-PET/MRI, which appears to have advantages over ^68^Ga-DOTATOC-PET/CT, especially with regards to abdominal lesions [6, 8]. Additionally, in the other studies same-session diffusion-weighed imaging was not available.

One limitation of this study is constituted by the heterogeneity of the patient population with regards to primary tumor, grading and treatment.

A further limitation of this study -besides the small sample size and its retrospective nature- was the segmentation method:

Tumor segmentation was performed semi-automatically whenever possible due to the higher degree of standardization; however, in some cases the faint tracer accumulation in hepatic lesions did not allow for the implementation of a threshold-based approach and a manual segmentation using the coregistered MRI was used.

A further limitation is that all patients in whom PRRT-related changes were assessed were previously and concomitantly treated with SSA. It is therefore not possible to assess the effect standalone PRRT has on tumor tissue. As some parameters, such as entropy are affected inversely by both treatments, the effect of PRRT might be masked by the additional SSA treatment.”

## Conclusion

Concluding we could not establish PET or ADC maps parameters that reliably predict treatment failure. When comparing patients responding to their current treatment protocol to patients showing treatment failure we observed some tendencies when it comes to changes in PET parameters, but statistical significance was not reached. The reasons for this remain unclear and further studies with larger sample sizes are warranted.

## Supplementary information

**Additional file 1: Figure S1.** Axial ADC maps (a + c) and PET (b + d) of 21 years-old patient with G3 NET of the pancreas and disease progression under chemotherapy. Interval between baseline (a + b) and follow-up (c + d) is 5 months.

**Additional file 2: Figure S2.** Axial ADC maps (a + c) and PET (b + d) of 64 years-old patient with G2 NET of the pancreas showing response to treatment with long-acting somatostatin analogues. Interval between baseline (a + b) and follow-up (c + d) is 12 months.

## Data Availability

Analyzed data are stored at the Department of Nuclear medicine, University clinic Essen.

## Abbreviations

NETs: Neuroendocrine tumors
SSTRs: somatostatin receptors
PET: positron-emission tomography
PRRT: radiopeptide therapy
ADC: apparent diffusion coefficient
SUVmax: maximum standardized uptake value
SUVmean: mean standardized uptake value
NENs: neuroendocrine neoplasias
CT: computed tomography
NECs: Neuroendocrine carcinomas
DW-MRI: diffusion-weighted MRI
PFS: progression-free survival
OS: overall survival
pNET: pancreatic NET
TFs: textural features
ROI: region of interest
SUVstd: standard deviation of the standardized uptake value
COV: coefficient of variance
TLRE: total lesion receptor expression
ADCmean: mean apparent diffusion coefficient
ADCstd: standard deviation of the apparent diffusion coefficient
ADCmax: maximum apparent diffusion coefficient
TLADC: total lesion apparent diffusion coefficient
e.g.: exempli gratia
etc.: et cetera
