# Supplementary material for: Treatment-related changes in neuroendocrine tumors as assessed by textural features derived from 68Ga-DOTATOC PET/MRI with simultaneous acquisition of apparent diffusion coefficient
Source: BMC Cancer. 2020 Apr 16;20:326. doi: 10.1186/s12885-020-06836-y (PMC7161278; doi:10.1186/s12885-020-06836-y)

Supplemental Figure 1:

Axial ADC maps (a+c) and PET (b+d) of 21 years-old patient with G3 NET of the pancreas and disease progression under chemotherapy. Interval between baseline (a+b) and follow-up (c+d) is 5 months.

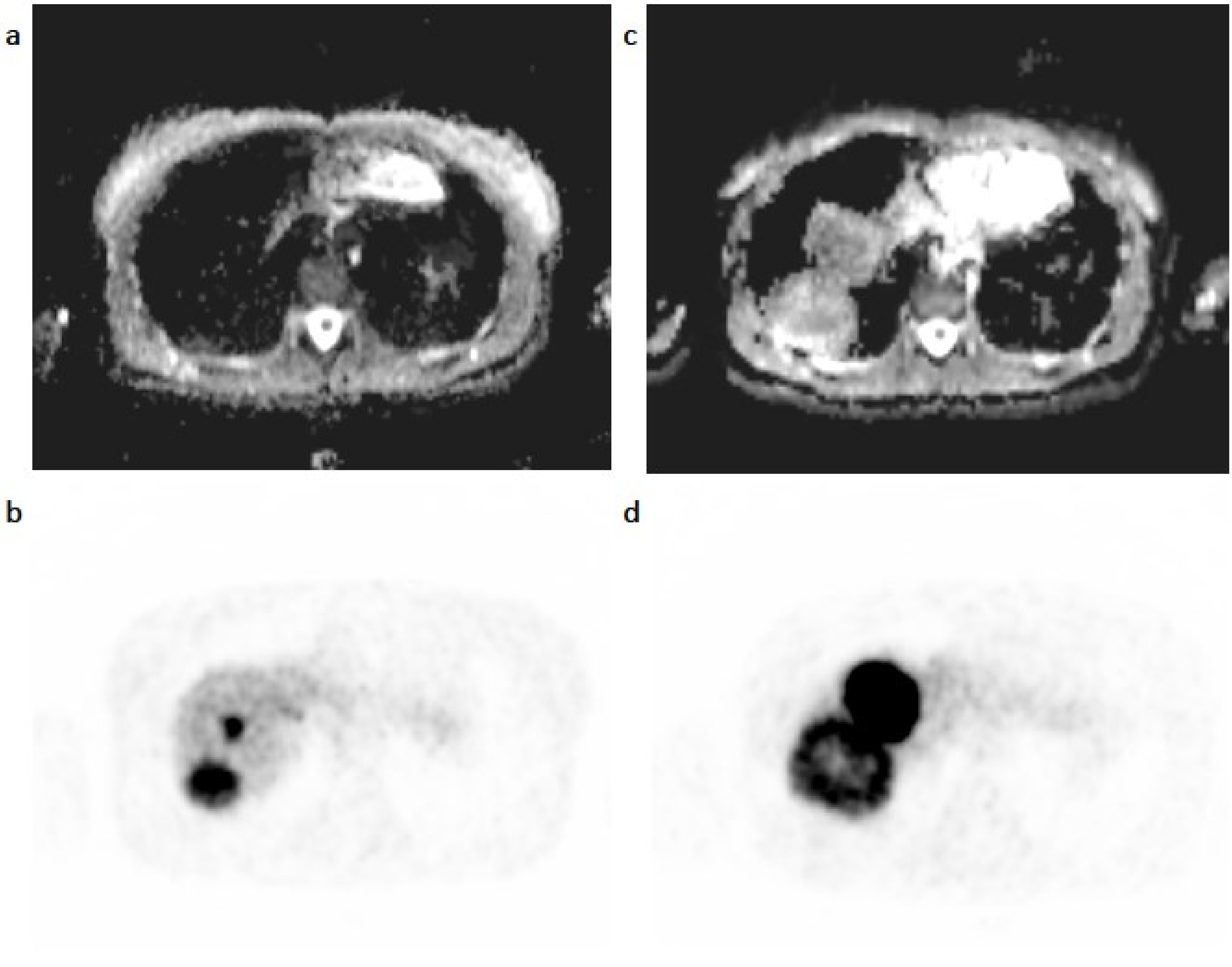

Supplement: Supplementary file 1 — Additional file 1: Figure S1. Axial ADC maps (a + c) and PET (b + d) of 21 years-old patient with G3 NET of the pancreas and disease progression under chemotherapy. Interval between baseline (a + b) and follow-up (c + d) is 5 months. [file 12885_2020_6836_MOESM1_ESM.pdf]
