# Supplementary material for: Treatment-related changes in neuroendocrine tumors as assessed by textural features derived from 68Ga-DOTATOC PET/MRI with simultaneous acquisition of apparent diffusion coefficient
Source: BMC Cancer. 2020 Apr 16;20:326. doi: 10.1186/s12885-020-06836-y (PMC7161278; doi:10.1186/s12885-020-06836-y)

Supplemental Figure 2:

Axial ADC maps (a+c) and PET (b+d) of 64 years-old patient with G2 NET of the pancreas showing response to treatment with long-acting somatostatin analogues. Interval between baseline (a+b) and follow-up (c+d) is 12 months.

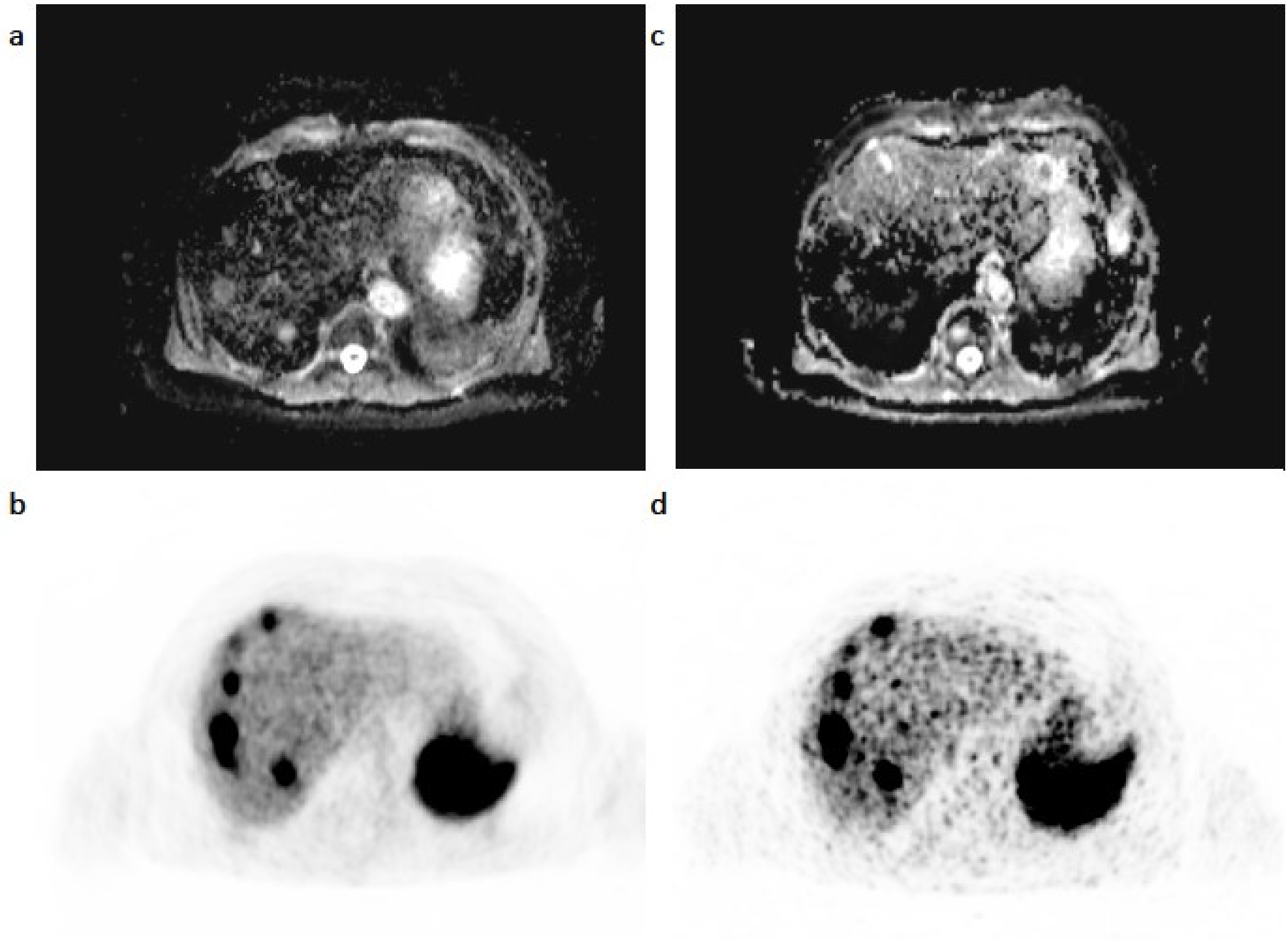

Supplement: Supplementary file 2 — Additional file 2: Figure S2. Axial ADC maps (a + c) and PET (b + d) of 64 years-old patient with G2 NET of the pancreas showing response to treatment with long-acting somatostatin analogues. Interval between baseline (a + b) and follow-up (c + d) is 12 months. [file 12885_2020_6836_MOESM2_ESM.pdf]
